# *Pontiella desulfatans* gen. nov., sp. nov., and *Pontiella sulfatireligans* sp. nov., Two Marine Anaerobes of the *Pontiellaceae* fam. nov. Producing Sulfated Glycosaminoglycan-like Exopolymers

**DOI:** 10.3390/microorganisms8060920

**Published:** 2020-06-18

**Authors:** Daan M. van Vliet, Yuemei Lin, Nicole J. Bale, Michel Koenen, Laura Villanueva, Alfons J. M. Stams, Irene Sánchez-Andrea

**Affiliations:** 1 Laboratory of Microbiology, Wageningen University and Research, Stippeneng 4, 6708 WE Wageningen, The Netherlands; dmvvliet@gmail.com (D.M.v.V.); fons.stams@wur.nl (A.J.M.S.); 2 Department of Biotechnology, Delft University of Technology, van der Maasweg 9, 2629 HZ Delft, The Netherlands; yuemei.lin@tudelft.nl; 3 Department of Marine Microbiology and Biogeochemistry, Royal Netherlands Institute for Sea Research (NIOZ) and Utrecht University, Landsdiep 4, 1797 SZ ’t Horntje (Texel), The Netherlands; nicole.bale@nioz.nl (N.J.B.); michel.koenen@nioz.nl (M.K.); laura.villanueva@nioz.nl (L.V.); 4 Centre of Biological Engineering, University of Minho, Campus de Gualtar, 4710-057 Braga, Portugal

**Keywords:** novel anaerobes, CAZymes, glycosaminoglycans, *Kiritimatiella*, Black Sea

## Abstract

Recently, we isolated two marine strains, F1^T^ and F21^T^, which together with *Kiritimatiella glycovorans* L21-Fru-AB^T^ are the only pure cultures of the class *Kiritimatiellae* within the phylum *Verrucomicrobiota.* Here, we present an in-depth genome-guided characterization of both isolates with emphasis on their exopolysaccharide synthesis. The strains only grew fermentatively on simple carbohydrates and sulfated polysaccharides. Strains F1^T^, F21^T^ and *K. glycovorans* reduced elemental sulfur, ferric citrate and anthraquinone-2,6-disulfonate during anaerobic growth on sugars. Both strains produced exopolysaccharides during stationary phase, probably with intracellularly stored glycogen as energy and carbon source. Exopolysaccharides included N-sulfated polysaccharides probably containing hexosamines and thus resembling glycosaminoglycans. This implies that the isolates can both degrade and produce sulfated polysaccharides. Both strains encoded an unprecedently high number of glycoside hydrolase genes (422 and 388, respectively), including prevalent alpha-L-fucosidase genes, which may be necessary for degrading complex sulfated polysaccharides such as fucoidan. Strain F21^T^ encoded three putative glycosaminoglycan sulfotransferases and a putative sulfate glycosaminoglycan biosynthesis gene cluster. Based on phylogenetic and chemotaxonomic analyses, we propose the taxa *Pontiella desulfatans* F1^T^ gen. nov., sp. nov. and *Pontiella sulfatireligans* F21^T^ sp. nov. as representatives of the *Pontiellaceae* fam. nov. within the class *Kiritimatiellae*.

## 1. Introduction

Sulfated polysaccharides are diverse and widespread. In animals, sulfated polysaccharides are present as sulfated glycan side-chains of mucin [1], and as sulfated glycosaminoglycans (mucopolysaccharides) such as chondroitin sulfate, an important component of cartilage [2]. Sulfated polysaccharides are prevalent in marine environments, where they are produced in high quantities by macroalgae, microalgae and bacteria [3]. This class of compounds forms an important substrate for marine microorganisms due to their prevalence. Degradation of sulfated polysaccharides involves the removal of sulfate groups by sulfatases [4]. Sulfatase genes are present in high numbers in the genomes of some marine bacteria of the *Planctomycetes-Verrucomicrobia-Chlamydiae* (PVC) superphylum such as *Rhodopirellula baltica* SH1^T^ [5] and *Lentisphaera araneosa* HTCC2155^T^ [6], supporting the idea that PVC bacteria are likely to be key degraders of sulfated polysaccharides in the marine environment. Anaerobic strains of sulfatase-rich PVC bacteria have rarely been studied thus far, despite the importance of anoxic marine sediments in the mineralization process [7].

In a previous study, we isolated two anaerobic marine bacteria from anoxic Black Sea sediment, strains F1^T^ and F21^T^, which grew on sulfated polysaccharides and belonged to the *Kiritimatiellae* class of the *Verrucomicrobiota* phylum [8]. This class was formerly known as *Verrucomicrobiota* subdivision 5 and initially proposed to represent a novel phylum [9]. *Kiritimatiellae* are widespread and abundant in anoxic environments such as the intestinal tract of vertebrate animals [10,11] and marine or hypersaline sediments [9,12]. Our marine isolates, strains F1^T^ and F21^T^, were found to represent a novel family-level *Kiritimatiellae* lineage [8] previously marked as ubiquitous yet uncultured (R76-B128) [13]. Currently, the only described species of the *Kiritimatiellae* class is *Kiritimatiella glycovorans* L21-Fru-AB^T^, a moderate halophile isolated from a hypersaline microbial mat [9]. Like several PVC bacteria described before [5,6,14,15], *K. glycovorans* was proposed to degrade sulfated polysaccharides *in situ* based on the presence of glycoside hydrolase and sulfatase genes in the genome, although stable growth could not be achieved *in vitro* [9]. In contrast, strains F1^T^ and F21^T^ were able to grow on complex sulfated polysaccharides, such as fucoidan, and notably encoded exceptionally high numbers of sulfatases (521 and 480, respectively) [8]. Here, we present a comprehensive characterization of both strains. Important physiological traits are revealed such as their ability to produce sulfated glycosaminoglycan-like exopolymers, a feature not yet described for bacterial pure cultures.

## 2. Materials and Methods

### 2.1. Strains, Growth Conditions and Substrates

Strains F1^T^ and F21^T^ were deposited at the German Collection of Microorganisms and Cell Cultures (DSMZ; Braunschweig, Lower Saxony, Germany) under the respective accession numbers DSM 106878^T^ and DSM 106829^T^, and at the Korean Collection for Type Cultures (KCTC; Jeongeup-si, South Korea) under the respective accession numbers KCTC 15641^T^ and KCTC 15642^T^. *K. glycovorans* L21-Fru-AB^T^ was ordered from the DSMZ (Braunschweig, Lower Saxony, Germany). Strains F1^T^ and F21^T^ were cultured in a basal anoxic bicarbonate-buffered marine medium described previously [8] containing 25 g L^−1^ NaCl for optimum salinity for growth, with 10 mM L-fucose as the substrate, unless mentioned otherwise. *K. glycovorans* was cultured in the same basal medium except for containing 60 g L^−1^ NaCl, with 5 mM D-glucose as the substrate. All cultures were incubated statically at 25 °C in the dark, unless mentioned otherwise. Substrates were obtained from Sigma-Aldrich (St. Louis, MO, USA), unless mentioned otherwise. Polysaccharide substrates were obtained from various distributors (Table 1).

### 2.2. Genome Annotation and Visualization

Genome sequencing, assembly and annotation with Prokka and InterProScan 5 was described previously [8]. Additionally, genes for the production of secondary metabolites (ectoine, pigments, potential antibiotics) were annotated with antiSMASH version 5.0 [16]. Peptidase genes were annotated by alignment with the MEROPS scan v12.0 database [17] using DIAMOND [18]. CAZymes were annotated with the dbCAN2 web server (HMMdb v7) [19]. Only hidden Markov model (HMM) matches were considered, and these were manually curated to exclude false positives. Genomes were visualized with CGView Server [20]. The visualization of inter-genome homology was based on a comparison of predicted proteins with a translated genome using blastx with an E-value cutoff of 10^−5^ and an identity cutoff of 30%. In general, encoded pathways for the degradation of substrates were explored through annotation with RAST v2.0 [21] and analysis and visualization with Pathway Tools v23.0 [22] and the MetaCyc database [23]. Cellular localization was predicted with SignalP [24] and PSORTb v3.0.2 [25]. Homologs of fucose degradation genes and glycosaminoglycan sulfotransferase genes were found by reciprocal matching of coding sequences. Blastp matches with protein sequences from literature were queried against the UniProtKB/Swiss-Prot database [26], and bitscores were compared to test reciprocity. Genome assemblies and Prokka annotations are available at the European Nucleotide Archive (ENA) through the sample accession numbers SAMEA5207384 and SAMEA5207385 for strain F1^T^ and strain F21^T^, respectively. The 16S rRNA gene sequences can be found with the respective accession numbers LS482847 and LS453290.

### 2.3. Physiological Tests

Analysis of respiratory quinones was carried out by the Identification Service and Dr. Brian Tindall of the DSMZ (Braunschweig, Lower Saxony, Germany). Catalase activity was tested by applying drops of 15% *v*/*v* hydrogen peroxide onto a pellet of active biomass obtained through centrifugation of 5 mL of a liquid culture (4700 × *g*, 10 min). Oxidase activity was tested with diagnostic oxidase strips (Merck, Darmstadt, Hesse, Germany). Gram staining was performed according to standard protocols and confirmed by applying a drop of 1 M NaOH solution onto a cell pellet, which leads to slimy wire formation within 10 s for Gram-negative cells. Cell size was deduced from phase contrast micrographs and scanning electron micrographs obtained as previously reported [8]. Growth in liquid culture was monitored through measuring optical density at 600 nm. The effect of salinity on growth was tested in triplicate 5 mL cultures in Hungate tubes with the NaCl concentration in the medium modified to 0%, 0.5%, 1%, 1.5%, 1.7%, 1.9%. 2.3%, 2.7%, 3.1%, 3.5%, 4.3%, 5%, 5.7% and 6.3%. The effect of pH on growth was also tested in triplicate 5 mL cultures buffered with 50 mM MES (pH 5, 5.5), PIPES (pH 6, 6.5, 7, 7.5) or Tris (pH 8, 8.5, 9). At pH values of 8.5 and 9, precipitation occurred. This could be avoided by a tenfold reduction in the added quantities of CaCl_2_, MgSO_4_ and MgCl_2_, but this inhibited growth in positive control cultures with a pH of 7. Therefore, absence of growth at pH > 8.5 is possibly due to the lack of Ca and/or Mg instead of pH effect. For ectoine analysis, strain F21^T^ was grown at the highest tolerated NaCl concentration (5% *w*/*v*). Ectoine was extracted by resuspending a cell pellet in 70% *v*/*v* ethanol and bead-beating the sample with mixed zirconia/silica beads of 2.5 and 0.5 mm diameter and a FastPrep bead-beater (MP Biomedicals, OH, USA) twice for 20 s at a speed setting of 6.0. Ectoine concentration was measured by high-pressure liquid chromatography (HPLC) using a Thermo Scientific Accela 600 HPLC equipped with an Agilent Polaris 3 NH2 column (100 × 4.6 mm) and a UV detector. The system was operated at 30 °C and 0.8 mL min^−1^ elution. The eluent was an isocratic mix of 75% *v*/*v* acetonitrile and 25% *v*/*v* Milli-Q water. Substrate tests for polysaccharides, organic acids, alcohols and H_2_/CO_2_ (80:20 *v*/*v*) were performed as described previously [8].

### 2.4. Reduction of External Electron Acceptors

The reduction of electron acceptors was tested in duplicate cultures with the following electron acceptors (concentration in mM): nitrate, 10; nitrite, 2; sulfite, 2; thiosulfate, 10; chemically produced elemental sulfur, 50; colloidal elemental sulfur, 50; biologically produced elemental sulfur (THIOPAQ^®^, Paques, Balk, The Netherlands), 50; dimethylsulfoxide (DMSO), 20; ferric citrate, 20; fumarate, 20; manganese oxide (MnO_2_), 10; MnO_2_/anthraquinone-2,6-disulfonate (AQDS), 10/0.1. Amorphous MnO_2_ was prepared from KMnO_4_ and MnCl_2_ as described by Burdige and Nealson [27]. Previously described methods were applied to measure nitrate and thiosulfate concentrations through anion chromatography, H_2_ partial pressures through gas chromatography (GC), concentrations of organic acids and alcohols through HPLC and dissolved sulfide concentration through the methylene blue colorimetric assay [8]. Dissolved dimethylsulfide produced by the reduction of DMSO was analyzed using a Thermo Scientific Accela 600 HPLC equipped with an Agilent Poroshell 120 EC-C18 column (4,6 × 250 mm; 4 µm) and a UV detector. The system was operated at 30 °C and 1 mL min^−1^ elution. The eluent was 0.1% *w*/*v* formic acid with a linear increase of 15% to 80% acetonitrile over the course of 12 min, followed by 80% acetonitrile for 5 min. Ferric and ferrous iron concentrations were quantified with the ferrozine assay [28]. To measure Mn(II) produced by the reduction of MnO_2_, the formaldoxime assay was used [29,30] after acidification of the samples to pH 1.5 with HCl [29,30].

### 2.5. Oxygen Gradient Cultures

To prepare oxygen gradient cultures, autoclaved Hungate tubes sealed with cotton plugs were filled with 10 mL anoxic basal medium containing 1% *w*/*v* SeaPlaque low-melting agarose (Lonza, Basel, Canton of Basel-City, Switzerland) and 5 mM L-fucose, and left to solidify. The final pH was 7.8. Media were inoculated with 5% *v*/*v* liquid culture and mixed well before dispensing into Hungate tubes. Phosphate salts were added from separately autoclaved anoxic stock solutions [31]. Oxidation of the medium was inferred from the color of the redox indicator resorufin, the product of the irreversible reduction of resazurin. Amplification, sequencing and analysis of full-length 16S rRNA gene sequences from culture samples was performed as described previously [8], with the purpose of verifying culture purity.

### 2.6. Energy Storage Compound Analysis

Cells were fixed for transmission electron microscopy (TEM) according to an adaptation of the protocol of Wittmann, et al. [32], as performed by Spring, et al. [9]. Shortly, biomass pellets were fixed in a fixative solution (5% *w*/*v* formaldehyde, 2% *w*/*v* glutaraldehyde) on ice for one hour, washed twice with washing buffer (0.1 M cacodylate), resuspended in 100 µL of phosphate-buffered gelatin and left to solidify for 20 min at 4 °C. Then, 500 µL of fixative solution was applied and the samples were incubated for 15 min at room temperature. The solid samples were cut into small pieces of around 0.2 mm^3^, fixed with fixation solution for another 30 min at room temperature and washed six times with washing buffer. Samples were then fixed with 1% *w*/*v* osmium tetroxide for 1 h at room temperature and washed three times with Milli-Q water. Then, samples were dehydrated with a graded series of acetone (10%, 30%, 50%, 70%, 90%, 100%) and embedded in Spurr epoxy resin as previously described [33]. Ultrathin sections were cut with a Leica EM UC7 ultramicrotome (Leica, Wetzlar, Hesse, Germany), and poststained using uranyl acetate and lead citrate. The sections were then examined on a JEOL JEM-1400 series 120kV TEM (JEOL, Tokyo, Japan). Cells were delicate, as many cells looked disintegrated, whereas *Desulfovibrio desulfuricans* G11 cells processed in parallel appeared mostly intact. Following the protocol for polyphosphate staining in bacterial cells by Havemeyer [34], cell pellets were stained with a 4′,6-diamidino-2-phenylindole (DAPI) solution of 1 µg mL^−1^, incubated for 30 min at room temperature, washed with phosphate-buffered saline and inspected with a BX41 fluorescence microscope (Olympus, Tokyo, Japan) equipped with an X-Cite Series 120Q metal-halide fluorescence lamp (Excelitas, Waltham, MA, USA), a 330–385 nm excitation filter and a 510–550 nm emission filter. As positive control, the staining of DNA by DAPI was inspected with the same excitation filter and a long-pass 420 nm emission filter.

### 2.7. Extracellular Polymeric Substances Analysis

For the analysis of exopolysaccharides, resazurin was omitted from the basal medium. The biomass was centrifuged at 10,000× *g* for 20 min. The extracellular polymeric substances (EPS) in the supernatant were precipitated by the addition of cold absolute ethanol to a final concentration of 50% (*v*/*v*). The precipitate was collected by centrifugation (10,000× *g*, 30 min), washed three times in absolute ethanol and lyophilized. Fourier-tra

nsform infrared spectroscopy (FTIR) spectra of the extracted EPS were recorded on an FTIR Spectrometer (PerkinElmer, Waltham, MA, USA) with a wavenumber range from 550 to 4000 cm^−1^. The lyophilized EPS was analyzed with scanning electron microscopy energy-dispersive X-ray (SEM-EDX) using a Philips XL 30 SEM (Philips, Amsterdam, The Netherlands). Prior to the SEM-EDX analysis, samples were metallized with gold and palladium. The sulfated polysaccharide content in the lyophilized EPS was measured with the Blyscan^TM^ assay (Biocolor, Carrickfergus, UK) following the manufacturer’s instructions. In brief, the sample (1.5 mg) was digested by papain extraction reagent overnight at 65 °C. After centrifugation at 10,000× *g* for 10 min, the supernatant was collected. Total sulfated polysaccharides were quantified by using the Blyscan^TM^ dye reagent containing 1,9-dimethylmethylene blue (DMMB) with bovine tracheal chondroitin 4-sulfate as the standard. In addition, the ratio of O- and N-sulfation of the sulfated polysaccharides was determined with the nitrous acid cleavage method. Nitrous acid reacts with N-sulfated hexosamine, cleaving off the sulfate ester group [35]. The difference between the total sulfated sites and the amount of O-sulfated sites after nitrous acid cleavage was used to determine the relative amount of N-sulfated hexosamine.

### 2.8. Phylogenetic Reconstruction

In May 2019, *Kiritimatiellales* genomes were retrieved for reconstructing phylogeny. Microbial Genomes Atlas (MiGA) [36] was used to query NCBI genomes, the metagenome-assembled genomes (MAGs) from Parks, et al. [37] and the Tara Oceans MAGs from Delmont, et al. [38]. NCBI genomes were also searched manually by taxonomic description. All selected genomes were >50% complete and <6% contaminated, with contamination occurring only in genomes with >80% completeness, as determined with CheckM v1.0.5 [39]. GTDB-Tk v0.2.2 was used to identify genes and generate a trimmed concatenated alignment [40], which was trimmed further with TrimAI v1.3 [41] using the “—gappyout” setting to a length of 4451 amino acid positions. Maximum-likelihood phylogeny was calculated with IQ-TREE v1.6.10 [42] using the LG+C30+F+G8 evolutionary model as selected by ModelFinder [43] from various possibilities. Branch support was determined with 1000 SH-like approximate likelihood ratio tests [44] and 1000 ultrafast bootstraps [45]. The tree was inspected with FigTree v1.4.2 (https://github.com/rambaut/figtree). Average amino acid identity (AAI) between genomes was calculated with the enveomics aai.rb script [46] using blastp.

### 2.9. Lipid and Cellular Fatty Acid Analysis

For the analysis of intact polar lipids (IPLs) and cellular fatty acids (CFAs), triplicate cultures of strains F1^T^ and F21^T^ and a culture of *K. glycovorans* were grown to early stationary phase at 20 °C with glucose (10 mM) as the substrate. The biomass was harvested by centrifuging at 10,000 × *g*, washed twice with 1.7% sterile saline solution and freeze-dried. In order to obtain CFAs, the freeze-dried biomass was hydrolyzed and derivatized as described previously [47]. Fatty acid methyl ester (FAME) quantification was carried out on an Agilent 7890B GC (Agilent, Santa Clara, CA, USA) with an Agilent CP Sil-5 silica column (25 × 0.32 mm) with gases, flow rate and oven temperature as described previously [47]. FAME identification was carried out on an Agilent 7890A GC coupled to an Agilent 5975C VL MSD mass spectrometer (MS) operated at 70 eV, with a mass range *m*/*z* 50–800 and 3 scans per second with the same column and oven settings as for the quantification. FAMEs were identified based on literature data and library mass spectra. Double bond positions were determined using dimethyldisulfide derivatization of the FAMEs as described previously [47]. IPLs were extracted from the freeze-dried biomass using a modified Bligh–Dyer procedure and analyzed through ultra-high pressure liquid chromatography-high resolution mass spectrometry (UHPLC-HRMS) as described by Bale, et al. [47]. IPLs were quantified in terms of their MS peak area response. As different IPLs show different response behavior, the relative abundance of the peak area does not necessarily reflect the actual relative abundance of the different IPLs. However, this method allows for a comparison between the strains analyzed in this study.

## 3. Results

### 3.1. Phenotypic Characterization

**Figure 1:**
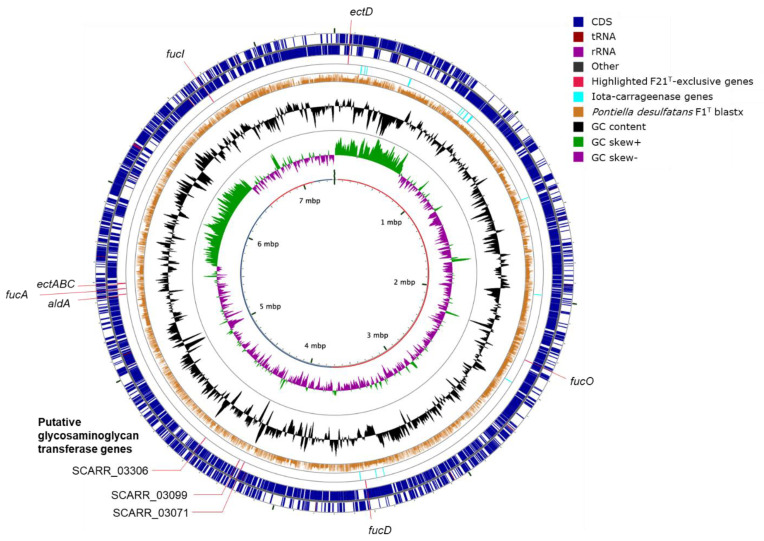
Circular visualization of the 7.4 Mbp strain F21^T^ genome. The inner circle shows the length of the genome in Mbp and distinguishes the contigs by an alternating red and blue color. Blastx hits with predicted proteins of strain F1^T^ are drawn with a height proportional to the amino acid identity percentage (0–100%). The outer ring shows coding sequences with a forward orientation, whereas the last-to-outer ring shows coding sequences with a backward orientation. Genes: *aldA*, lactaldehyde dehydrogenase; *ectABCD*, ectoine and 5-hydroxyectoine synthesis; *fucA*, L-fuculose phosphate aldolase; *fucD*, L-fuconate dehydratase; *fucI*, L-fucose isomerase; *fucO*, lactaldehyde reductase.

Strains F1^T^ and F21^T^ were neutrophilic and mesophilic cocci, stained Gram-negative and had a substrate range restricted to carbohydrates, similar to *K. glycovorans* (Table 2). However, unlike *K. glycovorans*, strains F1^T^ and F21^T^ were not oligotrophic and only slightly halophilic as defined by Ollivier, et al. [48]. Ammonium was required as nitrogen source, despite the presence of nitrogenase genes in the genomes (*nifDHK*, Table S1). The strains were capable of sulfate assimilation as demonstrated by sustained growth in sulfide-free cultures using ferrous iron as the reducing agent. This was in line with the presence of assimilatory sulfate reduction and sulfate transporter genes (*cysDNCHIJKM* and *sulP*, Table S1). Both strains lacked catalase activity despite encoding a catalase gene in their genomes (*katG*, Table S1). Strain F1^T^ showed no oxidase activity, whereas strain F21^T^ tested positive for oxidase. Yeast extract did not enhance the growth of the strains, and instead even slowed down the growth of strain F21^T^ (data not shown). Only strain F21^T^ showed psychrotolerance, as it was able to grow at temperatures as low as 0˚C. Additionally, strain F21^T^ was able to grow at higher salinity than strain F1^T^ (5.0% versus 3.1%, Figure S1). Strain F21^T^ produced ectoine (1 mg L^−1^ in a culture with an OD_600_ of 0.5), in line with the presence of the full ectoine biosynthesis pathway (*ectABC*, SCARR_04141-4143, Figure 1), which might explain its higher salt tolerance. Unlike strain F21^T^ cultures, cultures of strain F1^T^ had a yellow color. This may be due to the formation of lycopene, other carotenoids and/or aryl polyenes, as genes involved in their synthesis (*ctrBDQ*, *carA2*, *fabBFG*; Table S1) were encoded in the genome of strain F1^T^ but not in that of strain F21^T^.

### 3.2. Substrate Utilization and Genetic Capacity

Strains F1^T^ and F21^T^ showed growth on simple carbohydrates and sulfated polysaccharides (Table 2, Table S2). The sulfated polysaccharides supporting growth included four types of fucoidan formed by different macroalgae [8]. The strains did not grow on casamino acids, tryptone, yeast extract, L-alanine, L-aspartate, L-cysteine, L-glutamate or L-glycine [8], despite encoding respectively 83 and 70 peptidases, as well as amino acid transporters and amino acid degradation pathways. The strains did not show growth on H_2_/CO_2_, pyruvate, lactate, formate, acetate, propionate, butyrate, citrate, fumarate, malate, succinate, glycerol, methanol, ethanol, propanol, butanol or 1,2-propanediol. Growth on simple carbohydrates was in most cases consistent with the presence of degradation pathways and substrate transport genes in the genome (Table S2). However, the predicted ability to grow on D-mannose (strain F1^T^) and D-sorbitol (both strains) was not confirmed *in vitro*. Conversely, strain F1^T^ previously showed growth on D-tagatose and D-trehalose, but known genes for their degradation were not identified. Moreover, dedicated transporters for D-xylose, D-galacturonate and D-glucuronate were not identified, yet these compounds were utilized by both strains. Lastly, some genes of the fucose degradation pathway could not be identified (L-fuculokinase, *fucK*, both strains; lactaldehyde dehydrogenase, *aldA*, strain F1^T^).

The strains did not grow on any of the tested non-sulfated polysaccharides, such as agar, alginate, arabinan, cellulose, chitin, chitosan, laminarin, pectin, pullulan, starch, xanthan gum or xylan. Seemingly in contrast with this phenotype, exceptionally high numbers of carbohydrate-active enzymes (CAZymes) were encoded by strains F1^T^ and F21^T^ (540 and 514, respectively; Figure S2; Table S3). Most of these were glycoside hydrolase (GH) genes amounting to 422 and 388 genes, respectively accounting for 6.4% and 6.8% of all genes. Both strains encoded 59 different GH families. The most abundant GH genes were α-L-fucosidase genes (GH29, GH95, GH141) and genes of a polyspecific family (GH2). The strains also encoded fucoidanases (GH107), acetyl esterases (CE1-11), methyl esterases (CE15), chondroitin lyases (PL8) and a diversity of other GHs (Table S3). Strain F21^T^ possessed five α-2-*O*-methyl-L-fucosidase genes (GH139) [49], versus none in strain F1^T^. Strain F1^T^ encoded 56 α-L-rhamnosidase genes (GH28, GH78, GH106), whereas strain F21^T^ encoded only five. Their inability to degrade chitin, chitosan, alginate and pectin is in line with the absence of genes encoding the required hydrolytic enzymes (Table S3). However, they did have the genetic potential to degrade agar, arabinan, cellulose, laminarin, pullulan, starch and xylan. Taking agar as an example, agarose degradation to D-galactose and 3,6-anhydro-L-galactose requires β-agarase (GH50, 86), α-1,3-L-neoagarooligosaccharide hydrolase (GH117) and neoagarobiose hydrolase (GH117). Genes encoding enzymes of the according GH families were present in the genomes of our strains (Table S1), yet no growth on these substrates was observed.

### 3.3. Reduction of External Electron Acceptors during Anaerobic Growth on Sugars

**Figure 2:**
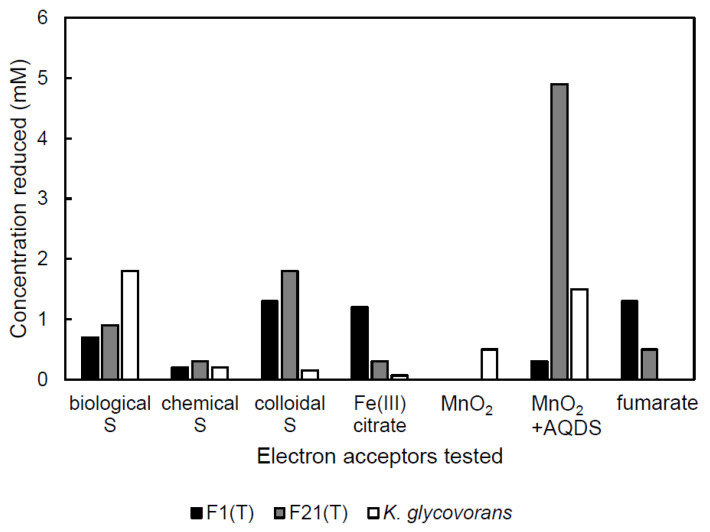
Concentration of external electron acceptor reduced during growth on sugars. Abbreviations: S, elemental sulfur; Fe(III), ferric iron; MnO_2_, manganese oxide; AQDS, anthraquinone-2,6-disulfonate.

Our two strains F1^T^ and F21^T^ as well as *K. glycovorans* reduced elemental sulfur, ferric citrate and fumarate when grown on their respective sugar substrates (Figure 2). In addition, dimethysulfoxide was reduced by strain F1^T^ (0.5 mM) and *K. glycovorans* (not quantified). Thiosulfate and nitrate were not reduced by any of the strains tested. The presence of sulfite or nitrite (2 mM) inhibited fermentative growth. While amorphous MnO_2_ was detectably reduced only by *K. glycovorans*, the addition of the electron shuttle and humic acid analogue AQDS (0.1 mM) stimulated MnO_2_ reduction in all the tested strains, particularly in strain F21^T^. Cultures of strain F21^T^ produced 4.9 mM of Mn(II) and about 75% less H_2_ than controls, equivalent to 14.9 kPa partial pressure or 6.1 aqueous mM difference. They produced 2.9 mM 1,2-propanediol versus 3.9 mM in controls. However, acetate production was unchanged at 7.2–7.3 mM. No growth was observed in transfer cultures with H_2_ as the electron donor and AQDS/MnO_2_ as the electron acceptors.

### 3.4. Response to Different Redox Conditions and Oxygen

**Figure 3:**
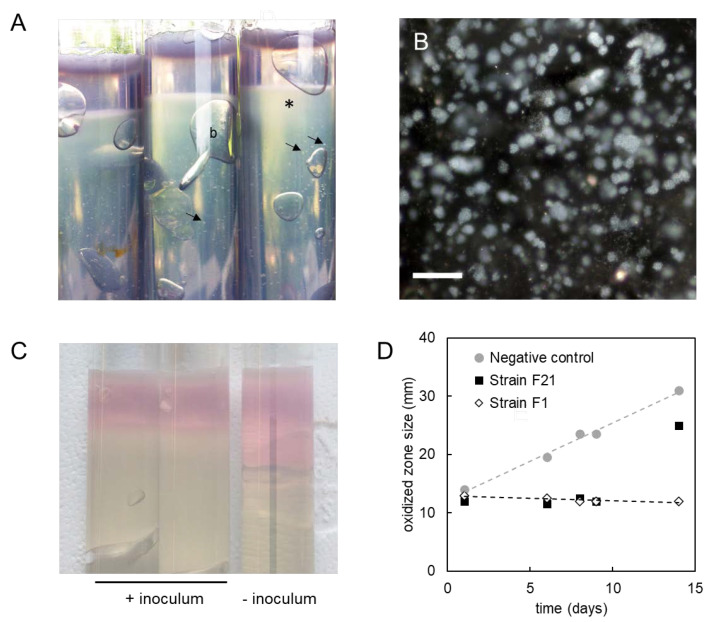
Growth of strains F1^T^ and F21^T^ in oxygen gradient cultures. (**A**) Cysteine-reduced cultures of strain F1^T^ after 19 days of incubation. Indicated are visible colonies (arrows), gas bubbles (b) and diffuse growth at the oxidized/reduced interface (*). (**B**) Micrograph showing the growth of microcolonies at the turbid oxidized/reduced interface. The scale bar represents a length of 100 µM. (**C**) Sulfide-reduced cultures after 8 days of incubation, of which two were inoculated with strain F21^T^ (left) and one was left uninoculated as the negative control (right). (**D**) Size of the pink oxidized zone versus incubation time in cultures reduced with sulfide. The negative controls were uninoculated. Plotted values are averages of two replicate cultures, which behaved reproducibly.

The strains grew in media with various reducing agents, such as cysteine (4 mM; E’^0^ = −0.22 V) or ferrous iron (2 mM; E’^0^ = 0 V). They also grew at higher redox potentials in the presence of ferric citrate (E’^0^ = 0.37 V) and amorphous MnO_2_ (E’^0^ = 0.47 V). Strains F1^T^ and F21^T^ did not grow in oxic media [8]. Strain F1^T^ was also incapable of growth in non-reduced liquid medium. In contrast, strain F21^T^ could grow in non-reduced medium and reduce it whilst doing so, although this ability was not completely reproducible among replicates. In oxygen gradient cultures, the strains grew only in the reduced zone (Figure 3A,B). Contaminations were ruled out by microscopical inspection of cell morphology and 16S rRNA gene amplicon sequencing. The reduced zone was larger in inoculated cultures than in uninoculated negative controls (Figure 3C). Its size remained stable for longer than a week, in contrast to the negative controls in which the diffusion of oxygen into the medium was visible as the oxidized zone enlarged over time (Figure 3D).

### 3.5. Formation of Energy Reserve Materials

**Figure 4:**
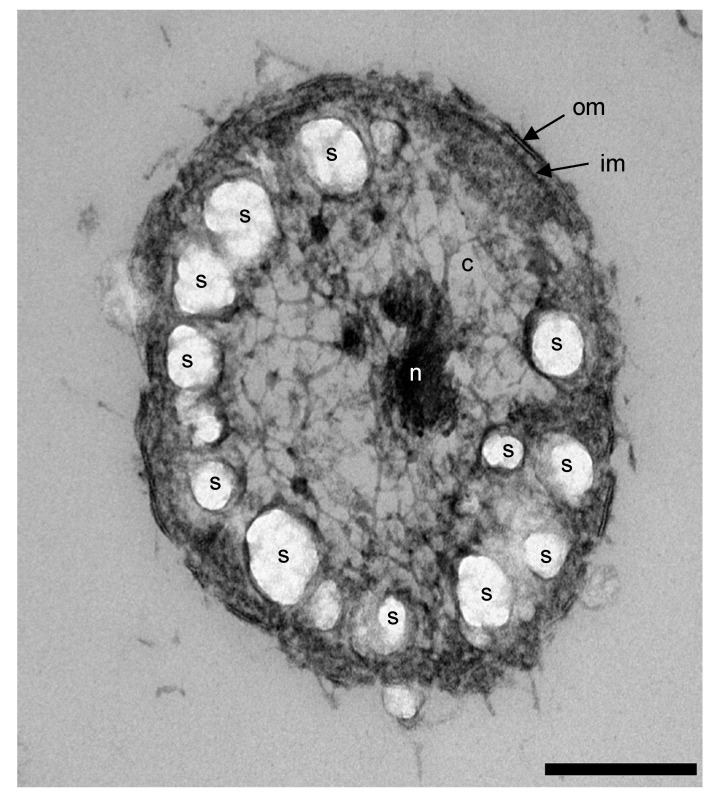
Transmission electron micrograph of a positively stained thin section of a *P. desulfatans* F1^T^ cell in late exponential phase, grown with glucose as the substrate. The scale bar corresponds to 200 nm. Arrows mark the outer membrane (om) and inner membrane (im). Further indicated are cytoplasm (c), nucleoid (n) and storage polymer granules (s).

In stationary phase, all fucose or glucose (up to 10 mM) was consumed by strains F1^T^ and F21^T^. The somewhat low electron recovery (strain F1^T^: 80%, strain F21^T^: 88%), not taking biomass into account [8], led us to hypothesize that the bacteria might form energy reserve materials. Microorganisms may store energy in granules of glycogen, polyphosphate and polyhydroxyalkanoates. Strains F1^T^ and F21^T^ showed the genetic potential for producing and using glycogen (*glgABCPX*, Table S1) and polyphosphate (*ppk, ppx*; Table S1), but not polyhydroxyalkanoates. Transmission electron microscopy of strain F1^T^ cells in exponential phase confirmed the presence of intracellular storage polymer granules (Figure 4), which appear electron-light since they do not stain with the applied osmium tetroxide or uranyl acetate [50,51,52]. A polyphosphate staining was negative for cells in exponential growth, indicating glycogen was probably the only energy storage compound formed in the conditions tested.

### 3.6. Production of Sulfated Glycosaminoglycan-like Exopolymers in Stationary Phase

**Figure 5:**
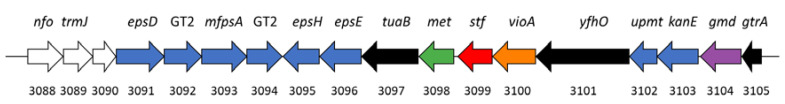
Putative sulfated glycosaminoglycan biosynthesis gene cluster in strain F21^T^. Locus tag numbers without the “SCARR_” prefix are indicated below the genes. Genes encoding glycosyltransferases are marked blue, auxiliary proteins marked black, methyltransferases marked green, sulfotransferases marked red, transaminases marked orange, dehydratases marked purple and genes of other or unknown function are marked white. Genes with the prefix *eps* are homologs with putative EPS biosynthesis glycosyltransferases from *Bacillus subtilis* strain 168. Abbreviations from left to right: *nfo*, apurinic endonuclease; *trmJ*, tRNA methyltransferase; *epsD*, GT4; *mfpsA*, mannosylfructose-phosphate synthase (GT4); *epsH*, GT2; *epsE*, GT2; *tuaB*, teichuronic acid biosynthesis protein; *met*, methyltransferase; *stf*, sulfotransferase; *vioA*, dTDP-4-amino-4,6-dideoxy-D-glucose transaminase; *yfhO*, bacterial membrane protein; *upmt*, undecaprenyl-phosphate mannosyltransferase (GT2); *kanE*, α-D-kanosaminyltransferase (GT4); *gmd*, GDP-mannose 4,6-dehydratase; *gtrA*, GtrA-like protein.

In stationary phase after growth on glucose or fucose, an increase in the viscosity of cultures was observed. Since the cells were intact under microscopical observation, we hypothesized the increased viscosity was not due to cell lysis but due to the production and release of extracellular polymeric substances (EPS). The spent medium supernatant contained sugars (approximately 50 µM), as determined with the anthrone assay with L-fucose as the standard. The presence of carbohydrate-based polymers in the supernatant was confirmed with Fourier-transform infrared spectroscopy (FTIR). The FTIR analysis indicated the presence of carbohydrates (a broad band at 3000–3600 cm^−1^, a strong band with the peak at 1080 cm^−1^) and sulfate substitutions (a shoulder band at 1230 cm^−1^), which was confirmed by scanning electron microscopy energy-dispersive X-ray analysis (File S1). The total sulfated polysaccharides in the EPS of strain F1^T^ and F21^T^ were 9 ± 1 and 11 ± 2 mg/g, respectively, based on the reaction with the 1,9-dimethylmethylene blue dye. Both pools of sulfated polysaccharide were primarily N-sulfated rather than O-sulfated, with 75% and 80% N-sulfation, respectively. A specific subclass of sulfated polysaccharides are sulfated glycosaminoglycans, defined as having a backbone of a hexosamine-containing repeating disaccharide, sulfated by a sulfotransferase [53]. Strain F21^T^ encoded three sulfotransferase genes with similarity to known glycosaminoglycan sulfotransferase genes (SCARR_03071, SCARR_03099 and SCARR_3306; Figure 1, Table S4). One of these putative glycosaminoglycan sulfotransferase genes (SCARR_03099) was located in a gene cluster containing eight potential hexosaminyltransferases (GT2, GT4) and a predicted dTDP-4-amino-4,6-dideoxy-D-glucose transaminase (Figure 5).

### 3.7. Phylogenomics and Chemotaxonomy of the Class Kiritimatiellales

**Figure 6:**
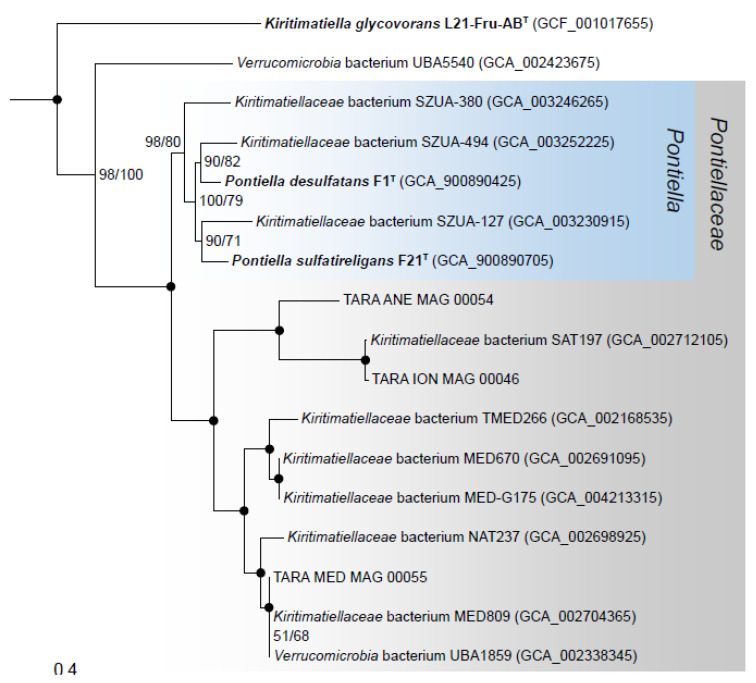
Maximum-likelihood phylogenetic tree of the members of the order *Kiritimatiellales* constructed from concatenated alignments of single-copy genes. Two *Kiritimatiellae* genomes outside of the *Kiritimatiellales* order (GTDB order UBA8416) were taken as outgroup and were omitted from the figure. Branch support is indicated with SH-like approximate likelihood ratio test values and ultra-fast bootstraps values, in that order. Black circles indicate support values of 100/100. The scale bar indicates substitutions per site. All cultured members are highlighted with bold font. The proposed novel genus and family are shaded in blue and grey, respectively. Since the UBA5540 and the SAT197 metagenome-assembled genomes (MAGs) share only 48% amino acid identity (AAI), we tentatively excluded MAG UBA5540 from the proposed novel family. MAG UBA5540 represents the uncultivated MSBL3 cluster based on the classification of its 16S rRNA gene using Silva ACT [56], and thus may represent an additional novel family within the *Kiritimatiellales*. NCBI accession numbers are indicated between parentheses, except for TARA oceans MAGs from Delmont, et al. [38] which can be accessed from https://doi.org/10.6084/m9.figshare.4902923.

In a concatenated single-copy gene phylogeny constructed with *Kiritimatiellales* genomes, strains F1^T^ and F21^T^ were placed in a monophyletic clade together with metagenome-assembled genomes (MAGs) from anoxic and oxic marine locations (Figure 6). The amino acid identity (AAI) of this clade with *K. glycovorans* was 44–50% (Table S5), close to the conservatively proposed AAI family-level threshold of 45% [54]. In contrast, the intra-clade AAI was >53%. The clade was congruent to a family-level clade (UBA1859) within the Genome Taxonomy Database (GTDB) [37]. Strain F1^T^ and F21^T^ shared an AAI of 73%, exceeding the 65% genus threshold [54]. Their digital DNA–DNA hybridization value was 24.5%, well below the species threshold of 70% [55].

Unlike *K. glycovorans*, strains F1^T^ and F21^T^ synthesized menaquinones (Table 2). Both strains produced MK-6, MK-7 and MK-8. Additionally, only strain F21^T^ produced MK-9, which also was the dominant menaquinone for this strain (55%). *K. glycovorans* and the isolates could also be distinguished by their cellular fatty acid (CFA) and intact polar lipid (IPLF) profiles (Table 2). While all three microorganisms produced *i*-C_14:0_, *i*-C_18:0_ and C_18:0_ as major CFAs, *i*-C_14:0_ was dominant in *K. glycovorans* (42%), whereas C_18:0_ was the most abundant CFA in strains F1^T^ and F21^T^ (40% and 35%, respectively). Additionally, the isolates contained a major fraction of *i*-C_12:0_. Only strain F21^T^ contained *i*-C_16:0_ as a major CFA. A detailed overview of CFAs can be found in Table S6. The major IPL classes observed in both strains F1^T^ and F21^T^ were phosphatidylglycerol (PG), monogalactosyldiacylglycerol (MGDG), cardiolipins and lyso-cardiolipins (Table 2). *K. glycovorans* had a similar IPL distribution, but in addition to PG, MGDG and the cardiolipins, two phosphoglycolipids were detected, confirming the previously reported detection of a phosphoglycolipid [9]. There were also low contributions from two unknown polar lipid components (Table S6). The phosphoglycolipids were further identified as phosphatidylglycerohexose (PG-Gly) based on a comparison of the tandem mass spectrometry fragmentation with published spectra [57,58] and based on the accurate mass of the PG-Gly lipids detected (Table S7).

## 4. Discussion

The various types of fucoidan are known for their heterogeneous compositions and complex structures [59]. The ability of strains F1^T^ and F21^T^ to grow on different types of fucoidan is thus consistent with the expansive CAZyme gene repertoires presented here and the reported sulfatase gene repertoires [8]. However, it should be noted that although the degradation of fucoidan by bacteria has been shown to involve fucosidases [60], fucoidanases [61,62,63], deacetylases [64] and sulfatases [65], there is currently no model of the exact enzymatic mechanism by which bacteria break down fucoidan into monomers. Various polysaccharides were tested as the substrate in this study, but only the sulfated ones were used by the isolated strains. However, the sulfated and non-sulfated polysaccharides tested also differ in backbone composition, implying other factors than sulfation could lead to the observed substrate profiles. To test the effect of polysaccharide sulfation on utilization by strains F1^T^ and F21^T^, the test should include non-sulfated fucoidan, carrageenan and chondroitin. Unfortunately, such compounds are not available commercially.

Strains F1^T^ and F21^T^ encoded the highest numbers of sulfatases, 521 and 480 sulfatases, respectively, reported for any described microorganism so far [8]. These numbers are exceptionally high, which can be best illustrated by a comparison with other bacteria that are known to contain a high number of sulfatase genes, such as *L. araneosa* HTCC2155^T^ (284 sulfatase genes) and *R. baltica* SH1^T^ (109 sulfatase genes). The research presented here has revealed similarly exceptional numbers of glycoside hydrolase genes (422 and 388, respectively). These exceed the numbers of GH genes predicted in *Bacteroidetes* spp.—which are regarded as important biopolymer degraders in marine and other environments [66,67,68]—such as *Bacteroides ovatus* ATCC 8483^T^ (324, [69]), *B. intestinalis* DSM 17393^T^ (319, [69]), *B. thetaiotaomicron* VPI-5482^T^ (286, [70]) and the marine *Zobellia galactanivorans* Dsij^T^ (141, [67]). The highest number of GH genes reported in the phylum *Verrucomicrobiota* is 261, encoded by *Victivallis vadensis* ATCC BAA-548^T^ [69]. Similar GH gene richness has only been found in fungi such as *Fusarium oxysporum* (396, [71]).

Both strains encoded putative carrageenan sulfatases [8] and potential kappa-carrageenases (polyspecific family GH16), but only strain F21^T^ encoded iota-carrageenases (GH82, Figure 1). In accordance, both strains were able to grow on kappa-carrageenan, but only F21^T^ grew on iota-carrageenan. The abundance of rhamnosidase genes in strain F1^T^, numbering 56, suggests its substrate range may include rhamnans. The GH gene profile of strain F1^T^ was similar to that of *Verrucomicrobiae* MAGs from a freshwater humic bog [72], implying the presence of similar compounds serving as substrates. Since strains F1^T^ and F21^T^ did not grow on amino acids or peptides, the many encoded peptidases may have a role in accessing the glycan chains of proteoglycans/glycoproteins through degradation of the peptide chains.

The ability to reduce external electron acceptors such as fumarate, elemental sulfur and ferric iron during fermentative growth on sugars is not unique to the three *Kiritimatiellales* spp. tested in this study. As an example, the reduction of elemental sulfur has also been observed for thermophilic archaea [73], several *Planctomycetes* [74,75,76] and the firmicute *Lucifera butyrica* [77], without a noticeable effect on the growth rate or yield. The *Kiritimatiellales* strains possessed no genes encoding proteins that could facilitate anaerobic respiration of sulfur compounds, such as polysulfide reductase or other types of molybdoenzymes [78], dissimilatory (bi)sulfite reductase pathway proteins or other reductive proteins listed by Wasmund, et al. [79]. Fumarate reduction was consistent with the presence of succinate dehydrogenase/fumarate reductase genes (*sdhABC*, Table S1). Nitrite inhibited growth, probably due to toxicity. However, the presence of cytochrome *c* nitrite reductase genes (*nrfAH*, Table S1) in the genomes of strains F1^T^ and F21^T^ suggests that the dissimilatory reduction of nitrite could occur at lower non-toxic concentrations of nitrite. AQDS is known to be reduced by lactic acid bacteria growing fermentatively, although not by *Escherichia coli* [80]. The reduction of metals during fermentative growth has been reported for members from various phyla [81,82]. The reduction of AQDS and metals can proceed through periplasmic or outer membrane c-type cytochromes that deliver electrons from quinones to these electron acceptors [83,84]. Although strains F1^T^ and F21^T^ did produce menaquinones and did encode multiple c-type cytochromes predicted to be localized in the periplasm or extracellularly (Table S1), *K. glycovorans* lacks both quinones and c-type cytochrome genes [9] yet still reduced AQDS and metals. It is thus unclear how and why these *Kiritimatiellales* reduce elemental sulfur, DMSO, AQDS and metals.

Strains F1^T^ and F21^T^ were shown to be intolerant to oxygen, contrasting the aerotolerance of *K. glycovorans*, which can grow fermentatively in oxic medium [9]. The strains showed resilient growth in anoxic cultures with heightened redox potential due to the addition of amorphous MnO_2_. Although the redox potential was not measured, the pink color of the redox indicator resorufin implied a redox potential higher than −0.02 to −0.03 V [85]. It is thus unclear if the inhibition of growth in non-reduced liquid cultures was due to the presence of trace oxygen, or due to a too high redox potential. Curiously, the oxygen gradient cultures showed a reduction of part of the oxidized zone and the prevention of oxidation over time (Figure 3). This could be explained by the reduction of oxygen, but also by a lowering of the redox potential by reduced fermentation products such as H_2_. As discussed in the previous paragraph, the strains are able to reduce external electron acceptors. The behavior in the oxygen gradient cultures could therefore be due to the reduction of such electron acceptors present in the medium, such as flavins, thiols or elemental sulfur (produced by sulfide oxidation), in turn causing the chemical reduction of oxygen. Such an oxygen reduction mechanism was demonstrated for the anaerobic, non-aerotolerant gut bacterium *Faecalibacterium prausnitzii* strain A2–165 [86]. Alternatively, the strains could reduce microaerobic levels of oxygen directly through the activity of cytochrome *bd* terminal oxidase (*cydAB*, Table S1) and/or cytochrome *cbb*_3_ oxidase (*ccoNOP*, Table S1), as previously proposed for the gut anaerobe *Akkermansia muciniphila* Muc^T^ [87]. Both oxidases have high affinity for oxygen and allow the conservation of energy through generating a proton motive force, although only cytochrome *cbb*_3_ oxidase is proton-pumping [88,89]. Strain F21^T^ showed oxidase activity when grown anaerobically, indicating a constitutive expression of at least one of these oxidase systems. Possibly, this constitutive expression enabled strain F21^T^ to grow in non-reduced liquid medium. Since the results from the oxygen gradient cultures are inconclusive, in-depth research as conducted for *F. prausnitzii* and *A. muciniphila* [86,87] is required to investigate whether oxygen is reduced, and if so, what mechanism is responsible. However, the results presented here—in combination with the multiple encoded putatively oxygen-dependent sulfatase maturation enzymes reported previously [8]—show that strains F1^T^ and F21^T^ could be adapted to proliferate under low oxygen concentrations.

Strains F1^T^ and F21^T^ produced EPS in the stationary phase, which is unusual but has also been reported for *L. araneosa* [90] and some other marine bacteria [91]. From our chemical and genomic analyses, we conclude that the EPS contained sulfated polysaccharides containing N-sulfated hexosamines, thus resembling sulfated glycosaminoglycans. Although the applied 1,9-dimethylmethylene blue assay is not specific for glycosaminoglycans and interacts with various sulfated polysaccharides [92,93], the high degree of N-sulfation in the sulfated polysaccharides detected here indicates the presence of sulfated hexosamines, such as found in the sulfated glycosaminoglycans heparin and heparan sulfate [53]. This is supported by the high number of predicted hexosaminyltransferases in the putative sulfated glycosaminoglycan biosynthesis gene cluster of strain F21^T^ (Figure 5). The detection of sulfated glycosaminoglycan-like compounds in the EPS of strains F1^T^ and F21^T^ is of fundamental microbiological importance. Sulfated glycosaminoglycans such as heparin/heparan sulfate and chondroitin sulfate are important components of animal tissues. Some prokaryotes are known to produce non-sulfated glycosaminoglycans [94,95] and sulfated exopolysaccharides [91], but little information concerning prokaryotes producing sulfated glycosaminoglycan-like polymers is available. Recently, sulfated glycosaminoglycan-like compounds have been detected in the extracellular matrix of granular sludge [96,97] and anammox granules [98], but it remains unclear which of the members of these microbial communities produced these compounds. The biosynthesis of sulfated glycosaminoglycans requires the sulfation of oligo- or polysaccharides, carried out by sulfotransferases [99]. While thoroughly studied in eukaryotes, sulfotransferases active towards glycosaminoglycans are currently not known to be encoded by prokaryotes. The identification of three putative glycosaminoglycan sulfotransferase genes and a putative sulfated glycosaminoglycan biosynthesis gene cluster (Figure 5) in strain F21^T^ corroborates the detection of sulfated glycosaminoglycan-like exopolymers. Additional research is needed to determine the structure and composition of the detected sulfated glycosaminoglycan-like exopolymers, and to identify the enzymes that synthesize them. 

Our chemotaxonomic investigations revealed the presence of PG-Gly lipids in *K. glycovorans*. These have been found previously in other halophiles, namely halophilic *Halomonas* bacteria [58] and extremely haloalkaliphilic *Natronobiforma cellulositropha* archaeal strains [57]. The production of PG-Gly lipids by *K. glycovorans* reinforces the association with halophiles, and lends further support to the hypothesis of Giordano, et al. [58] that PG-Gly lipids enhance the osmotic stability of the cellular membrane by increased steric protection through hydrogen bonding with lipid glycosyl headgroups. Our phylogenetic and chemotaxonomic results support the establishment of the novel taxonomic family *Pontiellaceae* fam. nov. These results are consistent with previous analyses, largely based on 16S rRNA genes [8]. Although these analyses have indicated that strains F1^T^ and F21^T^ represent two different genera, we now propose them as novel species of the genus *Pontiella* gen. nov. based on the phenotypic similarity and whole-genome comparison.

**Description of *Pontiellaceae* fam. nov.***Pontiellaceae* (Pon.ti.el.la.ce’ae. L. fem. dim. n. *Pontiella*, type genus of the family; suff. -*aceae*, ending to denote a family; L. fem. dim. pl. n. *Pontiellaceae*, the *Pontiella* family). Members of this family stain Gram-negative, and are found mainly in marine environments. The *Pontiellaceae* family corresponds phylogenetically to the R76-B128 clade as defined in the SILVA SSU r132 database and the UBA1859 family within GTDB taxonomy. It encompasses the type genus *Pontiella*, which contains two described species.

**Description of *Pontiella* gen. nov.***Pontiella* (Pon.ti.el’la. Gr. masc. adj. *pontios*, from the sea; L. fem. dim. n. *Pontiella*, she from the [Black] sea, referring to the origin of the type species). Stain Gram-negative. Non-motile and non-spore forming coccoid cells, which divide through binary fission. They produce menaquinones. Major cellular fatty acids are *i*-C_12:0_, *i*-C_14:0_, C_18:0_ and *i*-C_18:0_. Major intact polar lipid classes are phosphatidylglycerol, monogalactosyldiacylglycerol, cardiolipins and lyso-cardiolipins. Catalase activity is negative. Obligately anaerobic, mesophilic and neutrophilic. No yeast extract is required for growth, but at least 10 g L^−1^ NaCl is required. Growth occurs with simple carbohydrates and sulfated polysaccharides as the substrate. No dissimilatory reduction of nitrate or thiosulfate. Reduction of elemental sulfur, ferric citrate, fumarate and anthraquinone-2,6-disulfonate during fermentation. Sulfate is assimilated as a sulfur source when growing on glucose or fucose. Not aerotolerant. Produce and excrete exopolysaccharides, including sulfated glycosaminoglycan-like compounds. The type species is *Pontiella desulfatans*.

**Description of *Pontiella desulfatans* sp. nov.***Pontiella desulfatans* (de.sul.fa’tans. L. pref. *de*, off; N.L. masc. n. *sulfas –atis*, sulfate; N.L. part. adj. *desulfatans*, removing sulfate, referring to sulfate ester substitutions in polysaccharides). The genus description applies, with the following additional features. Cells have a diameter of 0.5–1.2 µm. Growth occurs at 10–30 °C, 10–31 g L^−1^ NaCl and a pH of 6.5–8.5. Optimal conditions are 25 °C, 23 g L^−1^ NaCl and pH 7.5. The following substrates are utilized: D-fructose, D-galactose, D-glucose, D-tagatose, D-trehalose, D-xylose, L-arabinose, L-fucose, L-rhamnose, D-cellobiose, D-lactose, D-maltose, D-sucrose, N-acetylglucosamine, D-glucuronate, kappa-carrageenan, chondroitin sulfate and fucoidan from *Cladosiphon* spp., *Fucus vesiculosus*, *Macrocystis pyrifera* and *Undaria pinnatifida*. The following compounds are not utilized: D-mannose, D-ribose, L-sorbose, raffinose, D-glucosamine, D-galacturonate, D-gluconate, D-galactitol, D-mannitol, D-sorbitol, myo-inositol, agar, arabinan, cellulose, laminarin, pullulan, starch, xanthan gum, xylan, chitin, chitosan, alginate, pectin, iota-carrageenan, casamino acids, tryptone, yeast extract, L-alanine, L-cysteine, L-glutamate, L-glycine, L-isoleucine, acetate, benzoate, butanol, butyrate, citrate, ethanol, formate, fumarate, glycerol, lactate, malate, methanol, propanol, propionate, pyruvate, succinate and H_2_/CO_2_. The main non-gaseous fermentation products from L-fucose are acetate and ethanol. The dominant menaquinone is MK-7. The type strain has a genome size of 8.6 Mbp and DNA G+C content of 56.0% (mol/mol). The type strain is F1^T^ (= DSM 106878^T^ = KCTC 15641^T^), isolated from anoxic Black Sea sediment.

**Description of *Pontiella sulfatireligans* sp. nov.***Pontiella sulfatireligans* (sul.fa’ti.re.li.gans. N.L. masc. n. *sulfas –atis*, sulfate; L. v. *religare*, to bind back, fasten up; N. L. part. adj. *sulfatireligans*, binding back sulfate, referring to sulfate ester substitutions in exopolymers). The genus description applies, with the following additional features. Cells have a diameter of 0.5–1.0 µm. Growth occurs at 0–25 °C, 10–50 g L^−1^ NaCl and a pH of 6.0–8.5. Optimal conditions are 25 °C, 23 g L^−1^ NaCl and pH 7.5. The following substrates are utilized: D-fructose, D-galactose, D-glucose, D-mannose, D-trehalose, D-xylose, L-fucose, L-rhamnose, D-cellobiose, D-lactose, D-maltose, D-sucrose, N-acetylglucosamine, D-galacturonate, D-glucuronate, D-mannitol, kappa-carrageenan, iota-carrageenan, chondroitin sulfate and fucoidan from *Cladosiphon* spp., *Fucus vesiculosus*, *Macrocystis pyrifera* and *Undaria pinnatifida*. The following compounds are not utilized: D-ribose, D-tagatose, L-arabinose, L-sorbose, raffinose, D-glucosamine, D-gluconate, D-galactitol, D-sorbitol, myo-inositol, agar, arabinan, cellulose, laminarin, pullulan, starch, xanthan gum, xylan, chitin, chitosan, alginate, pectin, Casamino acids, tryptone, yeast extract, L-alanine, L-cysteine, L-glutamate, L-glycine, L-isoleucine, acetate, benzoate, butanol, butyrate, citrate, ethanol, formate, fumarate, glycerol, lactate, malate, methanol, propanol, propionate, pyruvate, succinate and H_2_/CO_2_. The main non-gaseous fermentation products from L-fucose are acetate, ethanol and 1,2-propanediol. The cellular fatty acid *i*-C_16:0_ is produced in addition to the major cellular fatty acids in the genus description. The dominant menaquinone is MK-9. Ectoine is produced. The type strain has a genome size of 7.4 Mbp and DNA G+C content of 54.6% (mol/mol). The type strain is F21^T^ (= DSM 106829^T^ = KCTC 15642^T^), isolated from anoxic Black Sea sediment.

## Figures and Tables

**Table 1:** Polysaccharide substrates used in this study, their source or type, distributor and lot number, if mentioned on the packaging. “NR” stands for “not reported”.

Polysaccharide	Source/Type	Distributor	Lot Number
alginic acid	NR	Thermo Fisher Scientific (Waltham, MA, US)	NR
arabinan	sugar beet	Megazyme (Bray, Ireland)	80902b
cellulose	microgranular, CC41	Whatman (Maidstone, UK)	1441024
chitin	shrimp shells	Sigma-Aldrich (St. Louis, MO, US)	SLBL2694V
chitosan	shrimp shells	Sigma-Aldrich (St. Louis, MO, US)	BCBQ3414V
chondroitin sulfate	bovine trachea	Sigma-Aldrich (St. Louis, MO, US)	NR
laminarin	*Eisenia bicyclis*	abcr (Karlsruhe, Baden-Württemberg, Germany)	1025869
pectin	apple	Sigma-Aldrich (St. Louis, MO, US)	BCBK7271V
pullulan	*Aureobasidium pullulans*	Sigma-Aldrich (St. Louis, MO, US)	NR
starch	soluble	Sigma-Aldrich (St. Louis, MO, US)	SLBL2691V
xanthan gum	*Xanthomonas campestris*	Sigma-Aldrich (St. Louis, MO, US)	100M0218V
xylan	beechwood	Sigma-Aldrich (St. Louis, MO, US)	107H1209
κ-carrageenan	NR	Sigma-Aldrich (St. Louis, MO, US)	BCBR6980V
ι-carrageenan	NR	Sigma-Aldrich (St. Louis, MO, US)	SLBJ7874V

**Table 2:** Differential traits of strain F1^T^, F21^T^ and *Kiritimatiella glycovorans* L21-Fru-AB^T^. Abbreviations: MK, menaquinone; CL, cardiolipin; LCL, lysocardiolipin; MGDG, monogalactosyl diglyceride; PG, phosphatidylglycerol; PG-Gly, phosphatidylglycerohexose; +, positive; +/−, unstable, ceasing growth after the first transfer; −, negative; NDA, no data available. Major cellular fatty acids (CFAs), intact polar lipids (IPLs), quinones and fermentation products are reported in order of abundance. The CFA and IPL data for *K. glycovorans* were generated during this study, other data for *K. glycovorans* were obtained from Spring, et al. [9]. * Data from Van Vliet, et al. [8].

Species	*P. desulfatans*	*P. sulfatireligans*	*K. glycovorans*
Type Strain	F1^T^	F21^T^	L21-Fru-AB^T^
Isolation source	Anoxic marine sediment *	Anoxic marine sediment *	Hypersaline microbial mat
Cell diameter (μm)	0.5–1.2 *	0.5–1.0 *	1.0–2.0
Genome size (Mbp)	8.6 *	7.4 *	3.0
DNA G+C content (mol%)	56.0	54.6	63.3
Quinones	MK-7, MK-6, MK-8	MK-9, MK-8, MK-6, MK-7	none
Major CFAs (>5% of total)	C_18:0_, *i*-C_12:0_, *i*-C_14:0_, *i*-C_18:0_	C_18:0_, *i*-C_12:0_, *i*-C_18:0_, *i*-C_14:0_, *i*-C_16:0_	*i*-C_14:0_, C_18:0_, *i*-C_18:0_
Major IPLs	PG, LCL, CL, MGDG	PG, CL, MGDG, LCL	PG, CL, MGDG, PG-Gly, LCL
Oxidase activity	−	+	−
Temp. for growth (°C)			
Range	10–30 *	0–25	20–40
Optimum	25 *	25 *	28
NaCl conc. for growth (g L^−1^)			
Range	10–31	10–50	20–180
Optimum	23	23	60–70
pH for growth			
Range	6.5–8.5	6.0–8.5	6.5–8.0
Optimum	7.5	7.5	7.5
Substrate utilization			
Chondroitin sulfate	+ *	+ *	-
Fucoidan	+ *	+ *	+/-
Iota-carrageenan	− *	+ *	+/-
Arabinose	+ *	− *	−
Cellobiose	+ *	+ *	−
Fructose	+ *	+ *	−
Fucose	+ *	+ *	−
Galactose	+ *	+ *	+/−
Galacturonate	− *	+ *	NDA
Lactose	+ *	+ *	−
Maltose	+ *	+ *	−
Mannitol	− *	+ *	−
Mannose	− *	+ *	+
Rhamnose	+ *	+ *	+/−
Sucrose	+ *	+ *	−
Tagatose	+ *	- *	NDA
Trehalose	+ *	+ *	−
Major fermentation productsfrom L-fucose	Acetate, H_2_, ethanol, lactate *	Acetate, ethanol, H_2_, 1,2-propanediol *	−
Major non-gaseous fermentation products from D-glucose	Acetate, ethanol, lactate	Acetate, ethanol, lactate	Ethanol, acetate

## Supplementary Materials

The following are available online at https://www.mdpi.com/2076-2607/8/6/920/s1, File S1: FTIR and SEM-EDX results of EPS from strains F1^T^ and F21^T^, Figure S1: The effect of salinity on growth of strain F1^T^ and F21^T^, Figure S2: Circular visualization of the 8.7 Mbp strain F1^T^ genome, Table S1: Annotation of selected genes in strains F1^T^ and F21^T^ including a complete list of all CAZyme genes, Table S2: Utilization of saccharide and peptide substrates and presence of the corresponding degradation pathway genes and transporter genes in the genomes of strains F1^T^ and F21^T^, Table S3: Number of CAZyme genes detected in strains F1^T^ and F21^T^ per CAZyme (sub)family, Table S4: Sulfotransferase genes encoded by strain F21^T^ with a PF13469 sulfotransferase domain and their BlastP matches with studied sulfotransferases, Table S5: Amino acid identity matrix of *Kiritimatiellales* genomes, Table S6: CFAs and IPLs detected in strains F1^T^ and F21^T^ and *K. glycovorans*, Table S7: Accurate masses of the two detected IPLs with a phosphatidylglycerohexose (PG-Gly) head group.
