# Supplementary material for: Pontiella desulfatans gen. nov., sp. nov., and Pontiella sulfatireligans sp. nov., Two Marine Anaerobes of the Pontiellaceae fam. nov. Producing Sulfated Glycosaminoglycan-like Exopolymers
Source: Microorganisms. 2020 Jun 18;8(6):920. doi: 10.3390/microorganisms8060920 (PMC7356697; doi:10.3390/microorganisms8060920)
Supplement: Supplementary file 1 [file microorganisms-08-00920-s001.zip › Figure S2 - strain F1 circular genome figure.pdf]

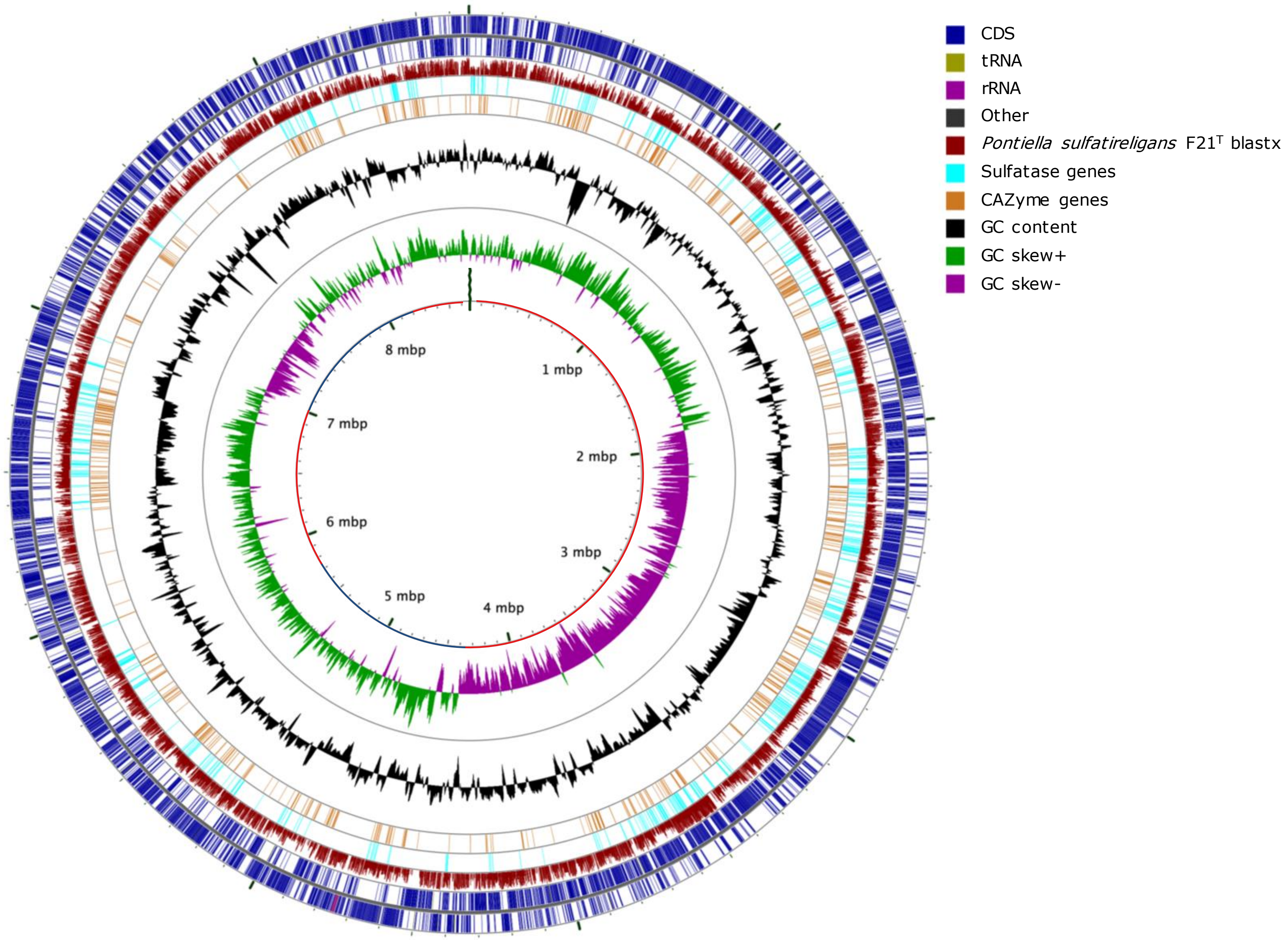

1 mbp
2 mbp
3 mbp
4 mbp
5 mbp
6 mbp
7 mbp
8 mbp
CDS
tRNA
rRNA
Other
Pontiella sulfatireligans F21^{T} blastx
Sulfatase genes
CAZyme genes
GC content
GC skew+
GC skew-
