# Supplementary material for: Pontiella desulfatans gen. nov., sp. nov., and Pontiella sulfatireligans sp. nov., Two Marine Anaerobes of the Pontiellaceae fam. nov. Producing Sulfated Glycosaminoglycan-like Exopolymers
Source: Microorganisms. 2020 Jun 18;8(6):920. doi: 10.3390/microorganisms8060920 (PMC7356697; doi:10.3390/microorganisms8060920)
Supplement: Supplementary file 1 [file microorganisms-08-00920-s001.zip › File S1 - FTIR and SEM-EDX.docx]

FTIR

SEM-EDX

# Strain F1^T^


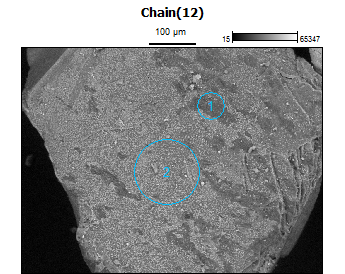


Image Name: Chain(12)

Image Resolution: 1024 by 768

Image Pixel Size: 0.62 µm

Acc. Voltage: 20.0 kV

Magnification: 200


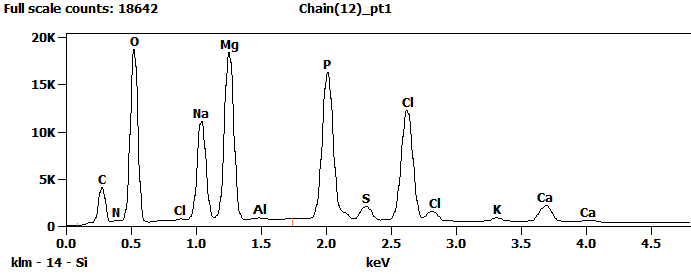


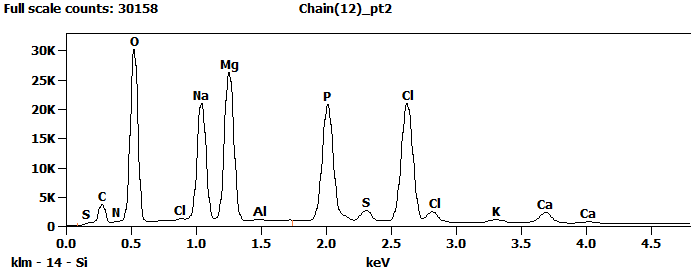


Net Counts

|  | ***C-K*** | ***N-K*** | ***O-K*** | ***Na-K*** | ***Mg-K*** | ***Al-K*** | ***P-K*** | ***S-K*** | ***Cl-K*** | ***K-K*** | ***Ca-K*** |
| --- | --- | --- | --- | --- | --- | --- | --- | --- | --- | --- | --- |
| ***Chain(12)_pt1*** | 22797 | 3168 | 110278 | 76988 | 136419 | 0 | 164968 | 16303 | 129744 | 5479 | 21622 |
| ***Chain(12)_pt2*** | 21205 | 3020 | 179149 | 150015 | 197521 | 0 | 211658 | 19446 | 226152 | 7986 | 22803 |

Weight %

|  | ***C-K*** | ***N-K*** | ***O-K*** | ***Na-K*** | ***Mg-K*** | ***Al-K*** | ***P-K*** | ***S-K*** | ***Cl-K*** | ***K-K*** | ***Ca-K*** |
| --- | --- | --- | --- | --- | --- | --- | --- | --- | --- | --- | --- |
| ***Chain(12)_pt1*** | 14.39 | 3.21 | 40.10 | 8.23 | 11.76 | 0.00 | 9.85 | 1.00 | 9.19 | 0.44 | 1.83 |
| ***Chain(12)_pt2*** | 10.28 | 1.98 | 41.29 | 11.08 | 12.38 | 0.00 | 9.05 | 0.84 | 11.28 | 0.45 | 1.37 |

Atom %

|  | ***C-K*** | ***N-K*** | ***O-K*** | ***Na-K*** | ***Mg-K*** | ***Al-K*** | ***P-K*** | ***S-K*** | ***Cl-K*** | ***K-K*** | ***Ca-K*** |
| --- | --- | --- | --- | --- | --- | --- | --- | --- | --- | --- | --- |
| ***Chain(12)_pt1*** | 22.02 | 4.21 | 46.07 | 6.58 | 8.89 | 0.00 | 5.84 | 0.57 | 4.77 | 0.21 | 0.84 |
| ***Chain(12)_pt2*** | 16.29 | 2.69 | 49.15 | 9.18 | 9.70 | 0.00 | 5.56 | 0.50 | 6.06 | 0.22 | 0.65 |

Formula

|  | ***C-K*** | ***N-K*** | ***O-K*** | ***Na-K*** | ***Mg-K*** | ***Al-K*** | ***P-K*** | ***S-K*** | ***Cl-K*** | ***K-K*** | ***Ca-K*** |
| --- | --- | --- | --- | --- | --- | --- | --- | --- | --- | --- | --- |
| ***Chain(12)_pt1*** | C | N | O | Na | Mg | Al | P | S | Cl | K | Ca |
| ***Chain(12)_pt2*** | C | N | O | Na | Mg | Al | P | S | Cl | K | Ca |

Compound %

|  | ***C*** | ***N*** | ***O*** | ***Na*** | ***Mg*** | ***Al*** | ***P*** | ***S*** | ***Cl*** | ***K*** | ***Ca*** |
| --- | --- | --- | --- | --- | --- | --- | --- | --- | --- | --- | --- |
| ***Chain(12)_pt1*** | 14.39 | 3.21 | 40.10 | 8.23 | 11.76 | 0.00 | 9.85 | 1.00 | 9.19 | 0.44 | 1.83 |
| ***Chain(12)_pt2*** | 10.28 | 1.98 | 41.29 | 11.08 | 12.38 | 0.00 | 9.05 | 0.84 | 11.28 | 0.45 | 1.37 |

# Strain F21^T^


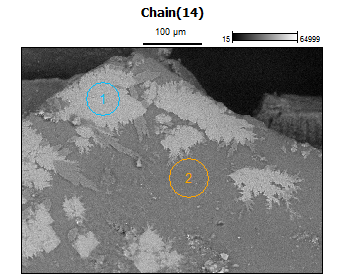


Image Name: Chain(14)

Image Resolution: 1024 by 768

Image Pixel Size: 0.49 µm

Acc. Voltage: 20.0 kV

Magnification: 250


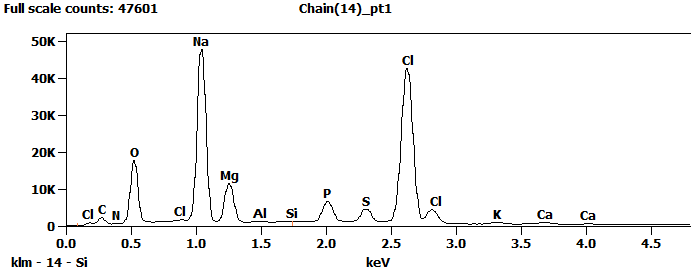


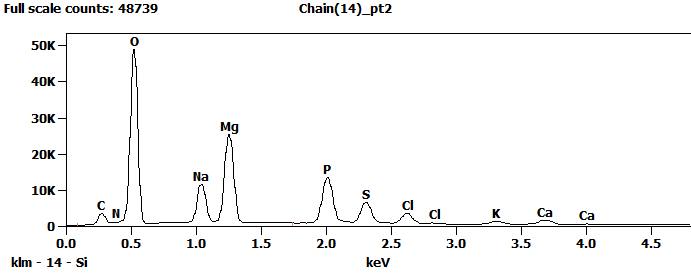


Net Counts

|  | ***C-K*** | ***N-K*** | ***O-K*** | ***Na-K*** | ***Mg-K*** | ***Al-K*** | ***Si-K*** | ***P-K*** | ***S-K*** | ***Cl-K*** | ***K-K*** | ***Ca-K*** |
| --- | --- | --- | --- | --- | --- | --- | --- | --- | --- | --- | --- | --- |
| ***Chain(14)_pt1*** | 12641 | 1096 | 100951 | 349464 | 80249 | 478 | 955 | 60639 | 43604 | 467593 | 5634 | 6119 |
| ***Chain(14)_pt2*** | 19872 | 4439 | 286317 | 80271 | 189894 |  |  | 135004 | 67224 | 32138 | 8637 | 14091 |

Weight %

|  | ***C-K*** | ***N-K*** | ***O-K*** | ***Na-K*** | ***Mg-K*** | ***Al-K*** | ***Si-K*** | ***P-K*** | ***S-K*** | ***Cl-K*** | ***K-K*** | ***Ca-K*** |
| --- | --- | --- | --- | --- | --- | --- | --- | --- | --- | --- | --- | --- |
| ***Chain(14)_pt1*** | 9.07 | 0.95 | 28.66 | 24.98 | 6.25 | 0.03 | 0.05 | 2.82 | 1.91 | 24.49 | 0.37 | 0.41 |
| ***Chain(14)_pt2*** | 7.78 | 2.24 | 55.68 | 7.43 | 13.67 |  |  | 6.65 | 3.27 | 1.84 | 0.53 | 0.93 |

Atom %

|  | ***C-K*** | ***N-K*** | ***O-K*** | ***Na-K*** | ***Mg-K*** | ***Al-K*** | ***Si-K*** | ***P-K*** | ***S-K*** | ***Cl-K*** | ***K-K*** | ***Ca-K*** |
| --- | --- | --- | --- | --- | --- | --- | --- | --- | --- | --- | --- | --- |
| ***Chain(14)_pt1*** | 15.66 | 1.40 | 37.15 | 22.53 | 5.33 | 0.02 | 0.04 | 1.89 | 1.24 | 14.33 | 0.19 | 0.21 |
| ***Chain(14)_pt2*** | 11.61 | 2.86 | 62.39 | 5.79 | 10.08 |  |  | 3.85 | 1.83 | 0.93 | 0.24 | 0.41 |

Formula

|  | ***C-K*** | ***N-K*** | ***O-K*** | ***Na-K*** | ***Mg-K*** | ***Al-K*** | ***Si-K*** | ***P-K*** | ***S-K*** | ***Cl-K*** | ***K-K*** | ***Ca-K*** |
| --- | --- | --- | --- | --- | --- | --- | --- | --- | --- | --- | --- | --- |
| ***Chain(14)_pt1*** | C | N | O | Na | Mg | Al | Si | P | S | Cl | K | Ca |
| ***Chain(14)_pt2*** | C | N | O | Na | Mg |  |  | P | S | Cl | K | Ca |

Compound %

|  | ***C*** | ***N*** | ***O*** | ***Na*** | ***Mg*** | ***Al*** | ***Si*** | ***P*** | ***S*** | ***Cl*** | ***K*** | ***Ca*** |
| --- | --- | --- | --- | --- | --- | --- | --- | --- | --- | --- | --- | --- |
| ***Chain(14)_pt1*** | 9.07 | 0.95 | 28.66 | 24.98 | 6.25 | 0.03 | 0.05 | 2.82 | 1.91 | 24.49 | 0.37 | 0.41 |
| ***Chain(14)_pt2*** | 7.78 | 2.24 | 55.68 | 7.43 | 13.67 |  |  | 6.65 | 3.27 | 1.84 | 0.53 | 0.93 |
