# Supplementary material for: Pontiella desulfatans gen. nov., sp. nov., and Pontiella sulfatireligans sp. nov., Two Marine Anaerobes of the Pontiellaceae fam. nov. Producing Sulfated Glycosaminoglycan-like Exopolymers
Source: Microorganisms. 2020 Jun 18;8(6):920. doi: 10.3390/microorganisms8060920 (PMC7356697; doi:10.3390/microorganisms8060920)
Supplement: Supplementary file 1 [file microorganisms-08-00920-s001.zip › Table S4 - Glycosaminoglycan sulfotransferases.docx]

**Table S4.** Sulfotransferases encoded by strain F21^T^ with a PF13469 sulfotransferase domain and their BlastP matches with studied sulfotransferases. The corresponding organism and UniProt accession numbers are indicated between parentheses. Glycosaminoglycan sulfotransferases are highlighted in bold.

| **locus tag** | **match** | **bitscore** | **query coverage (%)** | **identity (%)** |
| --- | --- | --- | --- | --- |
| **SCARR_03071** | 3-beta-hydroxysteroid sulfotransferase STD2 (*Cavia porcellus*, P52841) | 44 | 63 | 23 |
|  | Bile salt sulfotransferase SULT2A1 (*Homo sapiens*, Q06520) | 41 | 50 | 25 |
|  | **Heparan sulfate glucosamine 3-O-sulfotransferase 3B1** (*Homo sapiens*, Q9Y662) | 35 | 45 | 26 |
| **SCARR_03099** | Protein-tyrosine sulfotransferase A (*Caenorhabditis elegans*, O77081) | 72 | 79 | 23 |
|  | PAPS-dependent sulfotransferase Stf3 (*Mycobacterium tuberculosis* var. *bovis* AF2122/97, P64964) | 40 | 45 | 26 |
|  | Nodulation protein NoeE (*Sinorhizobium fredii* NGR234, P55472) | 39 | 49 | 26 |
|  | **Bifunctional heparan sulfate N-deacetylase/N-sulfotransferase 1** (*Homo sapiens*, P52848) | 29 | 2 | 54 |
|  | **Chondroitin sulfate GalNAc 6-O-sulfotransferase/carbohydrate sulfotransferase 15** (*Homo sapiens*, Q7LFX5) | 27 | 4 | 50 |
| **SCARR_03306** | Nodulation protein NoeE (*Sinorhizobium fredii* NGR234, P55472) | 40 | 82 | 22 |
|  | Protein-tyrosine sulfotransferase 2 (*Gallus gallus*, Q5ZJI0) | 40 | 82 | 21 |
|  | **Heparan sulfate glucosamine 3-O-sulfotransferase 6** (*Homo sapiens*, Q96QI5) | 36 | 10 | 44 |
|  | **Keratan galactose 6-O-sulfotransferase/carbohydrate sulfotransferase 1** (*Homo sapiens*, O43916) | 30 | 42 | 18 |
