# Supplementary material for: Pontiella desulfatans gen. nov., sp. nov., and Pontiella sulfatireligans sp. nov., Two Marine Anaerobes of the Pontiellaceae fam. nov. Producing Sulfated Glycosaminoglycan-like Exopolymers
Source: Microorganisms. 2020 Jun 18;8(6):920. doi: 10.3390/microorganisms8060920 (PMC7356697; doi:10.3390/microorganisms8060920)
Supplement: Supplementary file 1 [file microorganisms-08-00920-s001.zip › Table S7 - PGly detection.docx]

**Supplementary Table S7:** Accurate masses of the two detected IPLs with a phosphatidylglycerohexose (PG-Gly) head group. Total acyl carbons and double bonds equivalents show in brackets. AEC: assigned elemental composition, DAG: diacylglycerol, Δmmu: mass difference in milli mass unit (mmu), calculated with the formula [(*measured mass – calculated mass*) x 1000].

| **Intact polar lipid** | | **[M+NH_4_]^+^** | **AEC** | **Δmmu** |
| --- | --- | --- | --- | --- |
| **Polar head group** | **Core** |  |  |  |
| PG-Gly | DAG (32:0) | 902.5959 | C_44_H_89_O_15_NP^+^ | 0.5 |
| PG-Gly | DAG (34:1) | 928.6114 | C_46_H_91_O_15_NP^+^ | 0.7 |
