# Supplementary figures and images for: Pontiella desulfatans gen. nov., sp. nov., and Pontiella sulfatireligans sp. nov., Two Marine Anaerobes of the Pontiellaceae fam. nov. Producing Sulfated Glycosaminoglycan-like Exopolymers
Source: Microorganisms. 2020 Jun 18;8(6):920. doi: 10.3390/microorganisms8060920 (PMC7356697; doi:10.3390/microorganisms8060920)

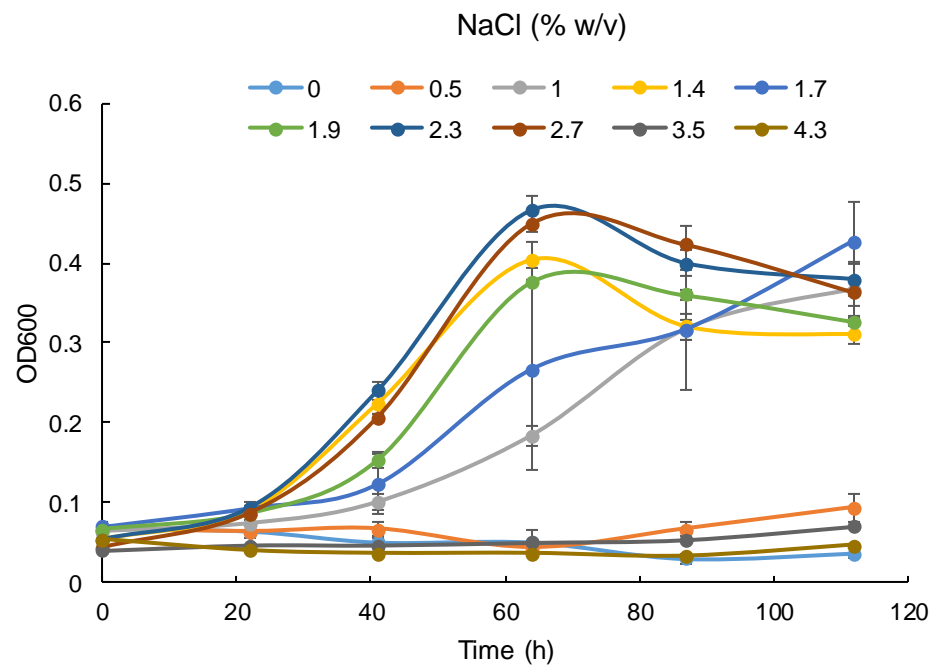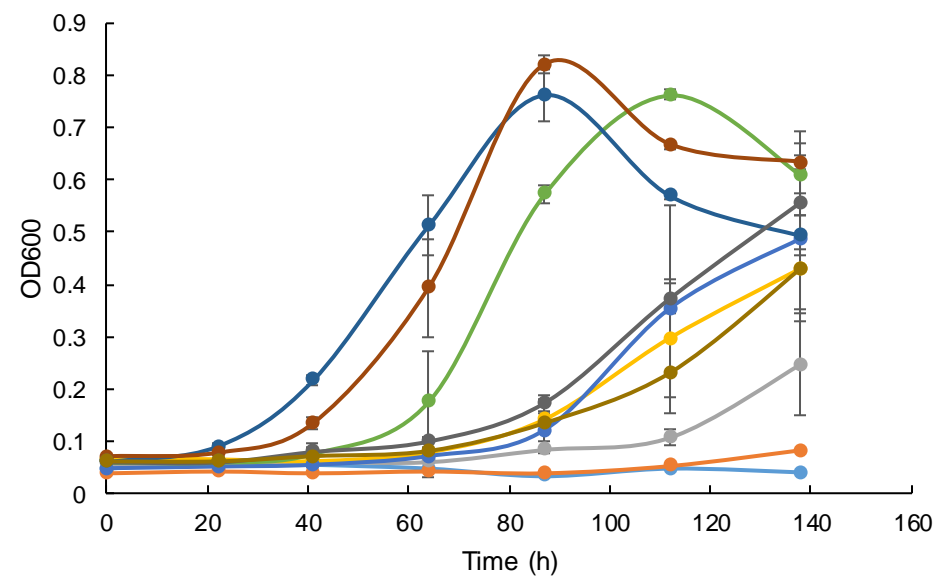

Supplement: Supplementary file 1 [file microorganisms-08-00920-s001.zip › Figure S1 - salinity growth curve figures.pdf]
